# Research progress on acupuncture treatment in central nervous system diseases based on NLRP3 inflammasome in animal models

**DOI:** 10.3389/fnins.2023.1118508

**Published:** 2023-02-28

**Authors:** Hai-Ming Zhang, Dan Luo, Rui Chen, Shu-Han Wang, Ya-Juan Zhao, Jin-Xiao Li, Min-Feng Zhou, Zhao-Min Yu, Jun-Li Zhang, Feng-Xia Liang

**Affiliations:** ^1^College of Acupuncture-Moxibustion and Orthopedics, Hubei University of Chinese Medicine, Wuhan, China; ^2^Department of Oncology, Integrated Traditional Chinese and Western Medicine, Central Hospital of Wuhan, Tongji Medical College, Huazhong University of Science and Technology, Wuhan, China; ^3^Department of Respiratory, Wuhan No.1 Hospital, Wuhan, China; ^4^Department of Integrated Traditional Chinese and Western Medicine, Union Hospital, Tongji Medical College, Huazhong University of Science and Technology, Wuhan, China; ^5^Department of Gastroenterology, Second Affiliated Hospital of Guangzhou Medical University, Guangzhou, China; ^6^Department of Oncology, Hubei Provincial Hospital of Integrated Chinese and Western Medicine, Wuhan, China; ^7^Division of Gastroenterology, Union Hospital, Tongji Medical College, Huazhong University of Science and Technology, Wuhan, China; ^8^Hubei Provincial Collaborative Innovation Center of Preventive Treatment by Acupuncture and Moxibustion, Wuhan, China

**Keywords:** acupuncture, central nervous system diseases, NLRP3, inflammasome, mechanism of acupuncture, neuroinflammation, anti-inflammation

## Abstract

Central nervous system (CNS) disorders exhibit complex neurophysiological and pathological mechanisms, which seriously affect the quality of life in patients. Acupuncture, widely accepted as complementary and alternative medicine, has been proven to exert significant therapeutic effects on CNS diseases. As a part of the innate immune system, NLRP3 inflammasome contributes to the pathogenesis of CNS diseases *via* regulating neuroinflammation. To further explore the mechanisms of acupuncture regulating NLRP3 inflammasome in CNS diseases, our study focused on the effects of acupuncture on neuroinflammation and the NLRP3 inflammasome in vascular dementia, Alzheimer’s disease, stroke, depression, and spinal cord injury. This study confirmed that the activation of NLRP3 inflammasome promotes the development of CNS diseases, and inhibiting the activation of NLRP3 inflammasome is a potential key target for the treatment of CNS diseases. In addition, it is concluded that acupuncture alleviates neuroinflammation by inhibiting the activation of the NLRP3 inflammasome pathway, thereby improving the progression of CNS diseases, which provides a theoretical basis for acupuncture to attenuate neuroinflammation and improve CNS diseases.

## Introduction

Central nervous system (CNS) diseases involve complex neuropathophysiological mechanisms, which seriously affect the quality of life in these patients, such as vascular dementia (VD), Alzheimer’s disease (AD), stroke, spinal cord injury (SCI), depression, Parkinson’s disease (PD), etc (Wei and Qiu, 2022). CNS diseases inevitably increase the global medical financial burden due to their high mortality rates, difficulty in diagnosis, and high treatment costs. Despite the high morbidity and enormous medical costs of CNS disorders, current treatment options are limited in that an insufficient understanding of the etiopathogenesis (Yu et al., 2022).

Inflammation is a defense response in the host system against pathogen infection and various tissue damage, and appropriate inflammation can remove the damage factors in time and enhance the body’s resistance to various pathogenic factors (Franceschi et al., 2018). Neuroinflammation is the key of CNS diseases. Many clinical and neuropathological studies have demonstrated that activated microglia, equivalent to macrophages in the brain and spinal cord, play a prominent role in the pathogenesis of neurodegenerative diseases as the first and most important line of immune defense in the CNS (Bellver-Landete et al., 2019). Excessive neuroinflammation leads to neuronal death and promotes the development of CNS diseases, while attenuated neuroinflammation is beneficial to neuronal survival and improves the symptoms and prognosis of CNS diseases (Dorothee, 2018). Numerous studies have shown microglia-mediated neuroinflammation is critical in CNS injury and prognosis.

Acupuncture is an effective therapeutic method, which has been widely practiced in China for more than 2,000 years. Several systematic reviews and meta-analyses have demonstrated that acupuncture can improve symptoms of CNS disorders, and its anti-inflammatory effects have long been proven (Chen et al., 2022b, He et al., 2022, Huang et al., 2022; Lin C. J. et al., 2022; Zhou Z. et al., 2022). Exhilaratingly, PROKR2 neurons are indispensable for the anti-inflammatory effect of low-intensity electroacupuncture (EA) through the vagal-adrenal axis, which was once again confirmed by the article published in the authoritative journal NATURE last year (Liu et al., 2021). In addition, EA is capable of regulating multiple cell signal transduction pathways to alleviate neuroinflammation (in animal models of stroke, AD, SCI, PD, and VD) (Chen et al., 2022b). The latest research found that EA inhibited the activation of microglia and polarized microglia to M2 phenotype, while EA reduced proinflammatory cytokines (IL-1β, TNF-α, and IL-6), increased anti-inflammatory cytokines (IL-4 and IL-10) (Xie et al., 2021). Although acupuncture, as an effective external treatment, has accumulated a lot of valuable experience in the practice of treating chronic diseases, the mechanism of acupuncture on inflammation and immune function is still unclear. Therefore, it is of great clinical significance to deeply study the mechanism of acupuncture anti-inflammation and immune regulation.

As a part of the innate immune system, nucleotide-binding oligomerization domain-like receptor protein 3 (NLRP3) inflammasome contributes to the pathogenesis of many diseases *via* regulating inflammation. Under physiological conditions, the NLRP3 inflammasome promotes efficient clearance of damaged cells and tissue repair through its dependent cytokines, thereby promoting tissue regeneration. At the same time, the NLRP3 inflammasome coordinates the invading pathogen-mediated immune responses and host-derived danger signals, maintaining the balance of pro-inflammatory and anti-inflammatory factors in the body. Under pathological conditions, the activation of NLRP3 inflammasome is related to the development of autoimmune diseases, which can lead to excessive inflammatory response. Recently, accumulating evidence has shown that the NLRP3 inflammasomes are activated in local tissues, the spinal cord, and brain regions in various animal models (Barczuk et al., 2022; Kou et al., 2022; Yao et al., 2022). At present, the exploration of the relationship between CNS diseases and NLRP3 inflammasome is still in its infancy, despite there being many studies on the relationship between CNS diseases and inflammatory factors. The activation of NLRP3 inflammasome mediates neuroinflammation to promote the progression of CNS diseases. Acupuncture improve of CNS diseases depends on inhibition of microglia-mediated neuroinflammation. Therefore, acupuncture targeting NLRP3 inflammasome to inhibit microglia mediated neuroinflammation is expected to become a decisive target to prevent CNS diseases. In this review, we summarized the new progress of acupuncture regulating the NLRP3 inflammasome activation-related signal pathway in the treatment of CNS diseases, which provides a theoretical basis for clarifying that acupuncture attenuates neuroinflammation and regulates immune cells.

## Sources and selection criteria

We searched Web of Science, PubMed, CNKI, and Embase. The search was limited to English or non-English articles with English abstracts published since the database’s inception to now. Keywords included (“acupuncture” or “electroacupuncture” or “EA”) and (“NLRP3” or “NLRP3 inflammasome” or “NOD-like receptor protein 3”) and (“stroke” or “vascular dementia” or “Alzheimer’s disease” or “depression” or “spinal cord injury” or “bulbar palsy” or “Parkinson’s disease” or “multiple sclerosis” or “traumatic brain injury” or “” or “brain tumor” or “cerebral palsy” or “headache” or “migraine” or “epilepsy” or “anxiety”). After being carefully evaluated, the information presented in the following studies was described and discussed.

## Acupoints for CNS diseases

**FIGURE 1:**
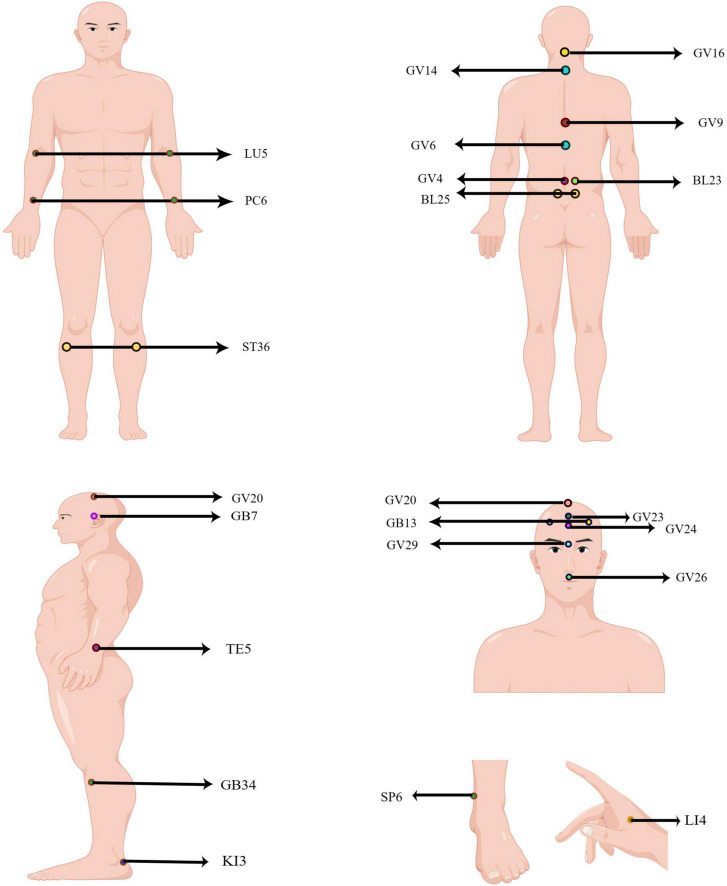
Human acupoints frequently used in CNS diseases. The locations of acupoint code are marked in the figure. GV20: Baihui; GV14: Dazhui; GV26: Shuigou; GV29: Yintang; GV24: Shenting; GB13: Benshen; GB7: Qubin; GV23: Shangxing; GV16: Fengfu; GV9: Zhiyang; GV6: Jizhong; GV4: Mingmen; ST36: Zusanli; GB34: Yanglingquan; BL23: Shenshu; LU5: Chize; LI4: Hegu; SP6: Sanyinjiao; TE5: Waiguan; PC6: Neiguan; BL25: Dachangshu; KI3: Taixi.

Acupuncture points (acupoints) are the “implicated acupoints” on the body surface during the pathological process of target organs, and the confirmed “implicated acupoints” are the parts of the body surface that play a “specific role” (Zhu, 2021). More specifically, acupoints are particular locations on the meridians, rich in nerves, blood vessels, and immune cells, which connect specific organs and regulate related body functions. Further study found that adenosine triphosphate (ATP) and transient receptor potential vanilloid (TRPV) channels were involved in acupuncture stimulation of acupoint regions (Lin J. G. et al., 2022). Based on the acupuncture theory, the selection of different acupoints has an essential impact on the efficacy of acupuncture in treating diseases, both in clinical and theoretical research. After analysis, the commonly used acupoints and the general rules of acupoint selection in CNS diseases were summarized. These acupoints include: Baihui, GV20; Dazhui, GV14; Shuigou, GV26; Yintang, GV29; Shenting, GV24; Benshen, GB13; Qubin, GB7; Shangxing, GV23; Fengfu, GV16; Zhiyang, GV9; Jizhong, GV6; Mingmen, GV4; Zusanli, ST36; Yanglingquan, GB34; Shenshu, BL23; Chize, LU5; Hegu, LI4; Sanyinjiao, SP6; Waiguan, TE5; Neiguan, PC6; Dachangshu, BL25; Taixi, KI3 (Figure 1).

## Relationship between acupuncture and NLRP3 inflammasome in CNS diseases

### Composition and activation of NLRP3 inflammasome

The NLRP3 inflammasome, a high molecular weight multiprotein complex of approximately 700 kDa, which consists of cytoplasmic NLRP3, apoptosis-associated speck-like protein (ASC) and pro-caspase-1, simplified as the receptor protein (NLRP3), adapter protein (ASC) and effector protein (caspase-1) (Swanson et al., 2019). NLRP3, as the core protein of NLRP3 inflammasome, is a pattern recognition receptor comprised of 11 leucine repeats at the C-terminal, NACHT domain in the middle, and Pyrin domain at the N-terminal. It functions in the cytoplasm to recognize exogenous microorganisms or endogenous danger signals and recruit the downstream connector protein ASC and the effector protein caspase-1. Specifically, when ASC binds to caspase-1, caspase-1 gathered on ASC splits at the junction of p20 and p10, thereby converting inactive pro-IL-1β and pro-IL-18 to mature active interleukin 1β (IL-1β) and interleukin 18 (IL-18) (Boucher et al., 2018).

**FIGURE 2:**
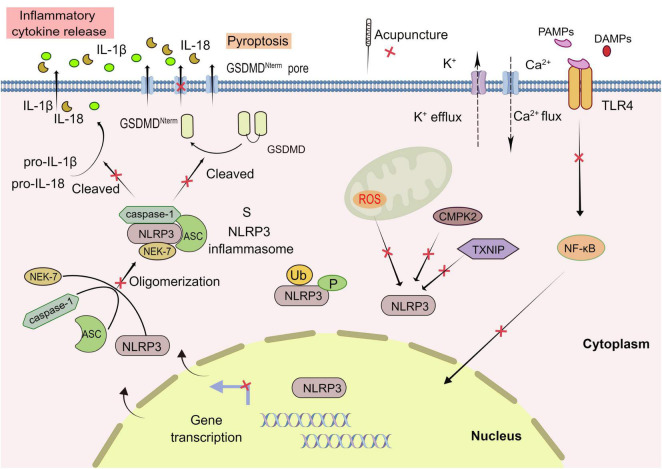
Acupuncture treatment in CNS diseases based on NLRP3 inflammasome. The activation of NLRP3 inflammasome involves the assembling of the components of NLRP3 inflammasome (NLRP3, ASC, caspase-1, and NEK7) to form a complete NLRP3 inflammasome complex. PAMPs, pathogen-associated molecular patterns; DAMPs, damage-associated molecular patterns; GSDMD, Gasdermin D; ROS, reactive oxygen species. Formation of the inflammasome activates caspase 1, which in turn cleaves pro-IL-1β and pro-IL-18. TLR, Toll-like receptor; NEK7, NIMA-related kinase 7; NF-κB, nuclear factor-κB; TXNIP, thioredoxin-interacting protein; CMPK2, cytosine monophosphate kinase 2; Ub, ubiquitylation; P, phosphorylation; Red X, represents a potential target for acupuncture treatment of CNS.

Currently, the mainstream view is that the activation of NLRP3 inflammasome requires two stages: priming and activation. In the priming stage, cytokines or pathogen-associated molecular patterns (PAMPs) activate Toll-like receptor (TLR), tumor necrosis factor (TNF), or nuclear transcription factor (NF-κB) signal pathway, to promote NLRP3 and upregulate pro-IL-1β mRNA expression (Bauernfeind et al., 2009). In the activation stage, the PAMPs and DAMPs (including sodium and potassium ion flow, active oxygen generation, mitochondrial dysfunction, etc.) result in the assembly and activation of NLRP3 inflammasome. Active caspase-1 cleaves gasdermin D (GSDMD) into a pore-forming N-terminal (GSDMD-N), which mediates the secretion of IL-1β and IL-18 as well as pyroptosis. Unlike apoptosis, pyroptosis is a proinflammatory programed cell death mode mediated by the inflammasome, characterized by cell swelling, lysis, and release of cell contents (Swanson et al., 2019; Figure 2).

### Acupuncture *via* NLRP3 inflammasome in the VD

VD is a clinical syndrome of cognitive dysfunction caused by cerebrovascular diseases, such as cerebral ischemia, cerebral hemorrhage, or brain hypoxia-ischemia (Yang et al., 2022). The main symptoms of VD are impairment of memory and executive ability. Although it has a high incidence rate, it is the only one among various types of dementia that can be prevented and treated early. The latest research has found that immune and neuroinflammation have gradually attracted the attention of researchers, excluding common risk factors for cerebrovascular diseases (Finger et al., 2022; Tian et al., 2022). Many studies have shown that acupuncture has a certain effect on VD (Chen et al., 2022b), but there are differences in efficacy due to different acupuncture methods, and there is no unified clinical standard. Scientific and reasonable selection of acupuncture methods is the key to acupuncture treatment of VD.

Thioredoxin-interacting protein (TXNIP) plays a vital role in oxidative stress (OS) and NLRP3 inflammasome activation. Manual acupuncture (MA) at ST36 and GV20 suppresses OS and inflammation by reducing TXNIP-mediated upregulation of hippocampal NLRP3 and IL-1β, thereby reducing cognitive impairment and neuronal death in VD rats (Du et al., 2018). Autophagy is a process of catabolism that relies on autophagy and lysosomes to degrade proteins, foreign bodies, and organelles to maintain the homeostasis of the internal environment. The study has found that electroacupuncture (EA) stimulation at GV20, GV14, and BL23 can improve the learning and memory ability of VD rats, reduce the ultrastructural damage of hippocampal CA1 neurons, and repair damaged neurons. It is speculated that the mechanism may be related to EA reducing the level of reactive oxygen species (ROS), the ratio of LC3-II/LC3-I, and inhibiting the expression of NLRP3 and beclin1 proteins, which potentially promotes the reduction of neuronal autophagy, inhibits the activation of the NLRP3 inflammasome, and attenuates the CNS inflammatory response (Qiu et al., 2022; Table 1).

**TABLE 1:** Acupuncture methods for VD, AD, and stroke *via* NLRP3 inflammasome.

Methods	Model	Tissue	Acupoints	Acupuncture strategy	Mechanisms	Animals	References
MA	VD	Hippocampal	ST36; GV20	MA were twisted 2 times per second for 30 s, once a day, 2 weeks, with a rest on the seventh day, total 12 treatments	TXNIP↓; SOD↓; ROS↓; NLRP3↓; ACS↓; caspase-1↓; IL-1β↓;	Wistar rats	Du et al. (2018)
EA	VD	Hippocampal	GV20; GV14; BL23	Dilatational wave, 10/50 Hz, 1 mA, 30 min, once a day, 4 weeks	NLRP3↓; LC3-II/LC3-I↓; beclin-1↓; ROS↓	SD rats	Qiu et al. (2022)
EA	CI	Hippocampal	ST36; GV20	Low frequency 2 Hz and high-frequency 10 Hz, 1 mA, 30 min, once a day, 14 consecutive days	NLRP3↓; caspase-1↓; ASC↓; IL-1β↓; IL-18↓; GSDMD↓; Aβ↓	SAMP8 mice; SAMR1 mice	Hou et al. (2020)
MA	HIE	Hippocampus	GB13	10 min, once a day, 14 consecutive days	NLRP3↓; ASC↓; caspase-1↓; IL-1β; IL-18↓	SD rats	Qu et al. (2021)
EA	CI	Hippocampus and colon	GV20; BL25; ST36	Continuous wave, 2 Hz, 1.0 mA, 15 min, once a day, 5 day a week, 5 weeks	NLRP3↓; TLR4↓; TNF-α↓; ASC↓; IL-1β↓; caspase-1↓; NF-κB p65↓; claudin-5; ZO-1	APP/PS1 mice	Liao et al. (2022)
EA	POCD	Hippocampus	GV20	2 Hz, 0.5 mA, 20 min, twice a day, 7 days	NLRP3↓; ASC↓; caspase-1↓; IL-1β↓; IL-6↓; NF-κB↓;	C57BL/6 mice	Sun et al. (2022)
EA	AD	Hippocampal	ST36; GV20	Continuous wave, 50 Hz, 1 mA, 20 min, once a day, 8 weeks	NLRP3↓; caspase-1↓; IL-1β↓	SD rats	He et al. (2020)
EA	AD	Hippocampus	GV20; GV26; GV29	Sparse wave, 2 Hz, 0.6 mA, 20 min, once a day, 15 days except on the 8th day	NLRP3↓; ACS↓; caspase-1↓; IL-1β↓	SAMP8 mice; SAMR1 mice	Jiang et al. (2018)
EA	AD	Hippocampus	GV20; GV24	2 Hz, 1 mA, 15 min, once a day, 5 consecutive days per week, 3 weeks	NLRP3↓; ASC↓; caspase-1↓; IL-1β; IL-18↓	PS cDKO mice	Li K. et al. (2021)
MA	Hemorrhagic stroke	Brain	GV20; GB7	Twist at the speed of 180–200 r/min, once every 5 min, with an interval of 5 min, 3 times in total, once every 12 h, for 7 days	NLRP3↓; IL-18↓; IL-1β↓	SD rats	Liu et al. (2020)
EA	Cerebral I/R model	Brain	LU5; LI4; SP6; ST36	Dilatational wave, 5/10 Hz, 2 mA, 20 min, 24 h	NLRP3↓; GSDMD↓; pro-caspase-1↓; cleaved-caspase-1 p20↓; pro-IL-1β↓; cleaved-IL-1β↓	C57BL/6 mice; Caspase-1 knockout mice	Cai et al. (2022)
EA	Stroke	Brain	GV20	Dilatational wave, 2/15 Hz, 1 mA, 30 min, once a day, five consecutive days	NLRP3↓; pro-caspase-1↓; caspase-1↓; pro-IL-1β↓; IL-1β↓; GSDMD↓; GSDMD-N↓	SD rats	Jiang et al. (2019)
EA	Stroke	Peri-infarct cortex	TE5; ST36	Continuous wave, 20 Hz, 1 mA, 30 min, once a day, seven consecutive days	NLRP3↓; miR-223↑; IL-18↓; caspase-1↓; IL-1β↓	SD rats	Sha et al. (2019)
EA	Stroke	Hippocampus	GV20; GV24	Sparse wave, 4 Hz, dense wave, 20 Hz, 0.5 mA, 30 min, once a day, 7 days	ROS↓; NLRP3↓; ASC↓; IL-18↓; caspase-1↓; LC3-II/LC3-I↓; IL-1β↓; Parkin↑; PINK1↑	SD rats	Zhong et al. (2022)

EA, electroacupuncture; MA, manual acupuncture; CI, cognitive impairment; HIE, hypoxic-ischemic encephalopathy; RIP3, receptor-interacting protein 3; POCD, postoperative cognitive dysfunction; SOD, superoxide dismutase; PS cDKO mice, PS1 and PS2 double knockout mice.

### Acupuncture *via* NLRP3 inflammasome in the AD

The condition of AD patients gradually deteriorates with age, manifested by various cognitive dysfunctions, including language impairment, disorientation, mood swings, loss of motivation, self-neglect, and behavioral abnormalities. Typical pathological features of AD are extracellular amyloid plaque, overexpression of tau protein and formation of nerve fiber tangle, accompanied by glial cell activation and neuroinflammation (Severini et al., 2021). A growing number of studies have shown that the NLRP3 inflammasome plays a vital role in the pathogenesis of AD by stimulating the innate immune response and activating the NLRP3 inflammasome (Ising et al., 2019; Moonen et al., 2022).

EA pretreatment on ST36 and GV20 acupoints can prevent learning/memory dysfunction in AD-like rats, the mechanism of which may be related to the down-regulation of hippocampal NLRP3, caspase-1, IL-1β protein expression, and inhibition of microglial activation (He et al., 2020). Moreover, EA at GV20 markedly preserved cognitive function in postoperative cognitive dysfunction (POCD) mice, associated with the inhibition of neuroinflammation as evidenced by reduced microglial activation and decreased IL-1β and IL-6 levels in brain tissue. Mechanistically, the activation of NLRP3 inflammasome and NF-κB was inhibited by EA, while the agonist of NLRP3 eliminates the therapeutic effect of EA on cognitive function. EA also preserved hippocampal neurons and tight junction proteins zonula occludens-1 (ZO-1) and claudin 5 (Sun et al., 2022). Besides, studies have confirmed that stimulating ST36 and GV20 acupoints with 2 Hz and 10 Hz EA ameliorates cognitive impairment. Intriguingly, cognitive function, hippocampal morphology, and TUNEL-positive cell counts were improved by stimulation with both EA frequency. Notably, 10 Hz EA was more effective than 2 Hz EA in reducing the number of TUNEL-positive cells in the CA1 area and serum IL-1β and IL-6 levels. Its mechanism may be that EA significantly reduced NLRP3, ASC, caspase-1, GSDM-D, IL-1β, and IL-18, but 2 Hz EA failed to effectively down-regulate the expression of ASC protein (Hou et al., 2020; Table 1).

### Acupuncture *via* NLRP3 inflammasome in the stroke

Stroke, a localized cerebral dysfunction caused by acute blood flow interruption, which includes 85% of ischemic stroke (manifested as focal infarction) and 15% of hemorrhagic stroke (manifested as cerebrovascular rupture), has been the leading cause of disability and death worldwide (Feigin and Krishnamurthi, 2016). The pathological progress of neuroinflammation in stroke requires the core involvement of the immune system. Ischemic stroke increases DAMPs levels, activating innate immune system sensors such as macrophages/microglia, neutrophils, and Toll-like receptors (TLRs) to co-amplify the inflammatory response (Lunemann et al., 2021). In addition, neuroinflammation induced by cerebral hemorrhage can release various inflammatory cytokines (such as IL-1β and IL-18), further aggravating the neuroinflammation (Anthony et al., 2022).

In recent years, mounting evidence has indicated that the inflammasomes have critical functions in inflammatory reactions and innate immunity. The NLRP3 inflammasome has been confirmed to be participated in brain injury after intracerebral hemorrhage (ICH) to mediate neuronal injury and neuroinflammation by serving as an important mediator of neuroinflammation after cerebral ischemia (Zhou et al., 2019). Inflammation, in particular, is one of the core pathological mechanisms of secondary injury of ischemic stroke (Chen et al., 2016). MA downregulates the expression of NLRP3, IL-1β, and IL-18 in the brain of ICH rats through GV20 to GB7 and inhibits the inflammatory response to promote the recovery of neurological function (Liu et al., 2020). Moreover, miR-223, a biomarker of multiple human metabolic ailments, was upregulated, while the levels of NLRP3, IL-1β, and caspase-1 decreased in the peri-infarct cortex of EA-treated rats with middle cerebral artery occlusion (MCAO). Nevertheless, the neuroprotective effect of EA was partially blocked by antagomir-223 (Sha et al., 2019).

EA ameliorates cognitive impairment by inhibiting NLRP3 inflammasome activation in stroke rats. EA at GV20 and GV24 attenuates cognitive impairment by regulating endogenous melatonin secretion through aralkylamine N-acetyltransferase gene synthesis in the pineal gland in MCAO rats and plays neuroprotective effects by upregulating mitophagy-associated proteins and suppressing ROS-induced NLRP3 inflammasome activation after ischemia-reperfusion injury (Zhong et al., 2022). Moreover, the results of Cai et al. (2022) revealed that the neuroprotective effect of EA is reflected in the inhibition of caspase-1 mediated neuronal pyroptosis and inflammatory response after cerebral ischemia/reperfusion. EA at LU5, LI4, SP6 and ST36 could decrease the score of neurological deficit, reduce the volume of cerebral infarction and improve the degree of nerve cell injury, and inhibit NLRP3, pro-caspase-1, cleaved-caspase-1 p20, pro-IL-1β, cleaved-IL-1β and GSDMD protein expression (Cai et al., 2022). This research shows that EA plays a neuroprotective role by interfering with the priming stage of NLRP3 inflammasome activation (Table 1).

### Acupuncture *via* NLRP3 inflammasome in the depression

Major depression is the most common mood disorder in China, with a lifetime prevalence of 3.4% and an annual prevalence of 2.1% (Huang et al., 2019). Conversely, it has a low treatment rate and few people receive proper treatment (Lu et al., 2021). In China, depression in elderly patients with chronic diseases leads to an increase in medical costs of 3.1–85.0% (Wu et al., 2022). EA can not only reduce the HAMD score, synergistically improve the efficacy of antidepressants, but also effectively reduce the side effects (Sun et al., 2013; Zhou Z. et al., 2022). In particular, the researchers found that GV20 and GV29 are the most commonly used acupoints for the treatment of depression. Given that the close relationship between neuroinflammation and depression has been widely confirmed (Troubat et al., 2021), it is speculated that neuroinflammation may be the critical therapeutic target for future depression treatment strategies. NLRP3 inflammasome is an intracellular multiprotein complex responsible for the innate immune processes associated with infection, inflammation, and depression. Subsequently, we analyzed how the inhibition of acupuncture on the activation of NLRP3 inflammasome alleviates depression.

The chronic unpredictable mild stress (CUMS) increases NLRP3 levels in the hippocampus. EA may improve the cognitive impairment of APP/PS1 mice by up-regulating the expression of claudin-5 and ZO-1, reducing the transposition of gut-derived lipopolysaccharide (LPS) to the CNS, inhibiting the over-activation of TLR4/NF-κB/NLRP3 pathway, and alleviating the inflammatory reaction of the CNS (Liao et al., 2022). Furthermore, EA stimulation of GV20, BL23 and KI3 acupoints inhibits the NF-κB/NLRP3 inflammasome pathway and improves CUMS-induced depressive behavior (Wang et al., 2022). In addition to EA, MA can significantly improve the depressive behavior of CUMS-induced rats at GV23 and GV16, the mechanism of which involves inhibiting the expression of NLRP3, ASC, caspase-1, IL-1β, IL-18, GSDMD, HMGB1, IFN-γ, IL-6, and TNF-α in serum and hippocampus. The above reports indicate acupuncture prevents CUMS-induced depression-like behaviors by reducing NLRP3-mediated pyroptosis and inflammatory responses (Chen et al., 2022a). Moreover, the antidepressant effect of acupuncture seems to be related to the inhibition of apoptosis in the prefrontal cortex (PFC). Acupuncture at GV20 and GV29 acupoints can reduce the number of TUNEL-positive cells and lower the protein expression of NLRP3, ASC coupled with caspase-1 in PFC (Wang H. M. et al., 2020; Li X. et al., 2021).

Other studies have found that patients with inflammatory bowel disease (IBD) are more susceptible to depression, with a prevalence rate of 33.1% (Gao et al., 2021). We all know, IBD is closely related to the activation of the NLRP3 inflammasome, inferring that NLRP3 is expected to become a new therapeutic target for IBD (Song et al., 2021). It was found that EA alleviated depression-like behavior in colitis model rats through their effects on the gut microbiome by modulating the hippocampal inflammatory response and metabolic disorders, as well as the hypothalamus-pituitary-adrenal (HPA) axis. EA at ST36 and SP6 not only significantly improved behavioral tests, but mechanistically it also upregulated the expression of ZO-1 and altered the composition of the gut microbiome (statistically increasing the density of producers of short-chain fatty acids such as *Ruminococcaceae*, *Phascolarctobacterium*, and *Akkermansiaceae*). Meanwhile, EA blocked the TLR4/NF-κB signaling pathways and NLRP3 inflammasome, along with downregulated the IL-1β level (Zhou F. et al., 2022; Table 2).

**TABLE 2:** Acupuncture methods for depression and SCI *via* NLRP3 inflammasome.

Methods	Disease	Tissue	Acupoints	Acupuncture strategy	Mechanisms	Animals	References
EA	Depression	Hippocampus	GV20; GB34	Dilatational wave, 2/100 Hz, 0.3 mA, 30 min, once every other day, 4 weeks	NLRP3↓; pro-IL-1β↓; IL-1β↓; ASC↓; IL-18↓; P2 × 7R↓; caspase-1↓; cleaved-caspase-1↓	SD rats	Yue et al. (2018)
MA	Depression	Hippocampus	GV23; GV16	20 min, once every other day, 28 days	NLRP3↓; IFN-γ; IL-6↓; ASC↓; IL-1β↓; caspase-1↓; IL-18↓; HMGB1↓; GSDMD↓; TNF-α↓	SD rats	Chen et al. (2022a)
EA	Colitis- related depression	Hippocampus	ST36; SP6	5 Hz, 0.2 mA, 30 min, once a day, 14 consecutive days	NLRP3↓; IL-1β↓; TLR4↓; NF-κB↓; p-NF-κB p65↓	SD rats	Zhou F. et al. (2022)
EA	Depression	Hippocampus	GV20; BL23; KI3	Sparse wave, 2 Hz, 0.6 mA, 15 min, once a day, 3 weeks	NLRP3↓; NF-κB↓; IL-6↓; IL-1β↓; IL-18↓; TNF-α↓	C57BL/6J mice	Wang et al. (2022)
MA	Depression	Prefrontal cortex	GV20; GV29	Twist at the speed of 60 r/min, 10 min, treatment 6 days per week, 6 weeks	NLRP3↓; ASC↓; IL-1β↓; caspase-1↓; IL-18↓	SD rats	Wang H. M. et al. (2020)
EA	SCI	Spinal cord	Jiaji (T9–T11)	2/100 Hz, 1 mA, 20 min, once a day, 1, 3, and 7 days	NLRP3↓; CMPK2↓; ASC↓; IL-1β↓; caspase-1↓; IL-18↓	SD rats	Chen et al. (2022c)
EA	SCI	Spinal cord	GV9; GV6	Continuous wave, 2 Hz, 30 min, once a day, 7 and 14 days	NLRP3↓; CGRP↑; ASC↓; caspase-1↓;	SD rats	Guo et al. (2021)

MA, manual acupuncture; EA, electroacupuncture; SCI, spinal cord injury.

### Acupuncture *via* NLRP3 inflammasome in the SCI

The traumatic event of SCI triggers a signaling cascade leading to glial activation, neuroinflammation, and neuron death. The activation of endocannabinoid receptor subtype 2 (CB2R) can reduce neuroinflammation by promoting the clearance of NLRP3, thus improving functional recovery of SCI. The mechanism of inhibiting neuroinflammation may be that CB2R promotes the differentiation of M2 macrophages/microglia, inhibits the differentiation of M1 macrophages/microglia, increases the expression of IL-10, and reduces IL-1β and IL-6 expression. In addition, activated CB2R also increases the ubiquitination of NLRP3, and interacts with autophagy related protein p62 and microtubule-associated proteins 1B light chain 3 (LC3) (Jiang et al., 2022). EA at GV14 and GV4 has the effect of promoting functional recovery after SCI and improving neuronal apoptosis. Furthermore, p38MAPK-mediated microglia activation and inflammatory reaction and JNK/p66Shc-mediated ROS generation and OS damage were both attenuated by EA. However, except for 50, 0.2, and 100 Hz EA fails to completely reverse the activation of microglia, apoptosis, inflammation, and the cascade of p38MAPK and NF-κB (Cheng et al., 2020).

Activation of NLRP3 is a vital mechanism of NLRP3 inflammasome activation and the inflammatory response following SCI. The dependence on cytosine monophosphate kinase 2 (CMPK2) catalytic activity provides opportunities for more effective control of NLRP3 inflammasome-associated diseases (Zhong et al., 2018). By constructing an adeno-associated virus (AAV) CMPK2 model to knock down the CMPK2 gene, Chen et al. found that EA at Jiaji (T9-T11) group and AAV CMPK2 group significantly improved the Basso Beattie Bresnahan score. EA and AAV CMPK2 group significantly reduced the protein expression levels of CMPK2, NLRP3, ASC, caspase-1, IL-18, and IL-1β, while the AAV CMPK2 blank group had the opposite results. In summary, CMPK2 promotes the expression of NLRP3, and EA downregulated the expression of CMPK2 and inhibited activation of NLRP3 inflammasome, which could elevate locomotion function in rats with SCI (Chen et al., 2022c). Furthermore, EA stimulation of GV9 and GV6 improves the locomotion of SCI rats, which is speculated to be inseparable from the up-regulation of calcitonin gene related peptide (CGRP) expression and the down-regulation of NLRP3, ASC, and caspase 1 expression in the spinal anterior horn tissue (Guo et al., 2021; Table 2).

## Discussion

Acupuncture is one of the traditional methods of treating CNS diseases in traditional Chinese medicine. It has the function of regulating qi and blood, and dredging the meridians. Its therapeutic effect on stroke, AD, VD, SCI, depression and other CNS diseases has been confirmed. According to our retrieval strategy, we searched the CNS diseases listed above through the database, and found that acupuncture regulating NLRP3 inflammasome mainly concentrated Alzheimer’s disease, vascular dementia, spinal cord injury, stroke and depression, and only one article about acupuncture treatment of Parkinson’s disease. For example, EA ameliorated dopaminergic neuron damage in PD rats through inhibiting NLRP3/Caspase-1 mediated neuronal pyrosis (Liu et al., 2022). Since there are few studies involving acupuncture to regulate NLRP3 inflammasome in the treatment of other CNS diseases, our study only discusses five diseases, including AD, VD, depression, stroke, and SCI.

### Acupuncture regulate microglia neuroinflammation in CNS diseases

With the development of modern advanced science and technology and scientists’ in-depth exploration of the physiological and biological mechanisms of acupuncture, so far, the mechanisms of acupuncture have involved central sensitization, neuroinflammation, neurotransmitters, immune regulation, oxidative stress, intestinal flora, etc. (Zhang et al., 2022). After integrating information in the brain, acupuncture modulates multiple neuroimmune pathways (including the vagus-adrenal medulla-dopamine, cholinergic anti-inflammatory, and sympathetic pathways, as well as the HPA axis) that ultimately act on immune cells by releasing crucial neurotransmitters and hormones (Li N. et al., 2021).

Microglia is an innate immune effector cell of the CNS, which plays the role of immune surveillance. Since microglia are the main source of inflammatory factors, neuroinflammation caused by its overactivation is the key to many CNS diseases. Further, activation of microglia and inflammation-mediated neurotoxicity are suggested to have essential roles in the pathogenesis of several neurodegenerative disorders. The activated microglia are classified into M1 and M2 phenotypes, which exert pro-inflammatory and anti-inflammatory effects, respectively. More and more studies have verified that acupuncture can inhibit neuritis by regulating the microglia phenotype, thereby improving or reversing the pathological process of CNS diseases (Wang L. et al., 2020; Li D. et al., 2021).

### Mechanism of acupuncture regulating NLRP3 inflammasome in CNS diseases

In CNS diseases animal models, such as AD, VA, depression, stroke and SCI, acupuncture regulated the NLRP3 inflammasome activation with the characteristics of multiple targets, multiple links and multiple pathways. In the priming stage, acupuncture reduced inflammatory factors (TLR4, TNF-α, IL-1β etc.) to inhibit the expression of NLPR3. In the activation stage, acupuncture constricted the oligomerization of NRLP3 inflammasome by lessening OS reaction and the expression level of each component of NLRP3 inflammasome (NLRP3, ASC, pro caspase-1). EA also decreased pro-caspase-1, caspase-1, pro-IL-1β and GSDMD by regulating the NLRP3 inflammasome mediated pyroptosis, inhibited the release of inflammatory factor (IL-1β, HMGB1, IL-18, INF- γ) and eventually mitigated neuroinflammatory response.

Additionally, EA diminished the level of ROS, the ratio of LC3-II/LC3-I, and inhibited the expression of NLRP3 and beclin 1 proteins, which potentially inhibited the activation of NLRP3 inflammasome. However, how EA regulated autophagy pathway and the interaction between autophagy and NLRP3 inflammasome need further study (Qiu et al., 2022). In addition, EA played a neuroprotective role by increasing miR-223 in the periinfarct cortex of MCAO rats to inhibit the levels of NLRP3, IL-1β and caspase-1 (Sha et al., 2019). Acupuncture suppressed TXNIP-mediated NLRP3 and IL-1β in VD rats (Du et al., 2018). EA downregulated the expression of CMPK2 and restrained activation of NLRP3 inflammasome, which could restore locomotion function in rats with SCI (Chen et al., 2022c). Moreover, EA downregulated of NLRP3, ASC, and caspase-1 expression in the spinal anterior horn tissue by raising CGRP expression (Guo et al., 2021).

It was noteworthy that the therapeutic effects of EA with different frequencies were different. 10 Hz EA was more efficient than 2 Hz EA in reducing the number of TUNEL positive cells in CA1 area of SAMR1 mice. The potential reason was that 10 Hz EA significantly reduces NLRP3, ASC, caspase-1, GSDM-D, IL-1 β, and IL-18 levels, but 2 Hz EA failed to effectively reduce the expression of ASC protein (Hou et al., 2020). However, excepted for 50, 0.2, and 100 Hz EA failed to completely reverse the activation of microglia, apoptosis, inflammation, and the cascade of p38MAPK and NF-κB (Cheng et al., 2020).

### Regulation of NLRP3 inflammasome

The activation of NLRP3 inflammasome is often accompanied by a variety of regulatory mechanisms. Ubiquitination of NLRP3 exerts a bidirectional regulatory role in activating the NLRP3 inflammasome, depending on the type of ubiquitin ligase and ubiquitination. Moreover, phosphorylation of NLRP3 may have a reaction to the activation of inflammasome, but the specific mechanism is unclear. The activation process of NLRP3 inflammasome is also related to the role of other proteins. For example, TXNIP can activate NLRP3 inflammasome after interacting with NLRP3 as an oxidation sensor. CMPK2, belonging to the nucleotide kinase family, can activate NLRP3 and play a crucial role in chronic inflammatory diseases. In turn, activation of the NLRP3 inflammasome relies on the catalytic activity of CMPK2, which provides a target for more effective control of NLRP3 inflammasome-associated diseases (Zhong et al., 2018).

Significantly, it has been found that all stimuli to NLRP3, whether or not they induce K^+^ efflux or NLRP3 mutations, require NIMA-related kinase-7 (Nek7) to activate the NLRP3 inflammasome. Thus, as a proximal regulator of NLRP3 oligomerization and an assembly component of the NLRP3 inflammasome, Nek 7 is a crucial regulatory molecule during the activation (He et al., 2016; Swanson et al., 2019). Besides, DDX3X is also a protein necessary for stimulating NLRP3 inflammasome (Samir et al., 2019). To sum up, since it involves a myriad of factors, the activation process of NLRP3 is sophisticated and indeterminate, which depends on in-depth studies to elaborate on the exact regulatory principles.

## Summary and future directions

This article summarized the role of NLRP3 inflammasome in a variety of common CNS diseases, expounded on its critical activation and regulation mechanisms, and detailed how acupuncture can improve CNS diseases *via* regulating the activation of NLRP3. With the development of immunological research, it has become increasingly clear that NLRP3 plays an indispensable role in different CNS diseases. Neuroinflammation is a cascade of immune responses mediated by innate immune residents of the CNS that can be triggered by damaging processes such as ischemia and hypoxia (Sha et al., 2021). In one respect, neuroinflammation maintains the stability of the microenvironment, but it can also cause damage to brain cells and neurons due to excessive activation of the inflammatory response (Uddin et al., 2020; Dhapola et al., 2021). Despite its importance in infections and sterile tissue damage, the exact mechanisms controlling and enabling NLRP3 activation are still being elucidated. Diverse cellular perturbations trigger NLRP3 activation, including the disruption of cellular ion homeostasis, lysosomes, and mitochondrial function or metabolism. However, how these perturbations relate to each other and how they converge on a common molecular mechanism that activates NLRP3 remain ongoing areas of research. Deeply clarifying the role of NLRP3 inflammasome in CNS diseases will lay a foundation for comprehensively understanding the occurrence and development of diseases and proposing specific targeted treatment methods with NLRP3 inflammasome inhibitors as the core.

Up to now, the research on the mechanism of acupuncture has been conducted by means of analysis and reduction. It is undeniable that the research method with reductionism as the main body has brought us a lot of new knowledge in the exploration of the mechanism of acupuncture, as well as a series of reliable evidence for the scientific nature of acupuncture therapy. However, due to the complexity of individual, as well as the bidirectional, multi-level and multi-target nature of acupuncture regulation, it is difficult for the research method with reductionism as the main body to give a complete answer to the relevant mechanism. Although some studies have provided partial evidence for EA in remedying CNS diseases, there is still a lack of high-quality randomized controlled trials to ascertain the efficacy and safety of EA in CNS diseases from an overall level. MA differs from EA with electrical stimulation signals. Many studies have shown that the frequency and intensity of acupuncture will produce different therapeutic effects. Future research directions, it is suggested, further explore the frequency, intensity and duration of MA or EA on CNS diseases.

## Author contributions

H-MZ, RC, and F-XL conceived the main ideas. H-MZ, J-LZ, and DL wrote the initial manuscript. J-LZ and F-XL revised and edited the manuscript. S-HW and Y-JZ designed the framework. M-FZ and J-XL helped search the references. Z-MY helped illustrate the figures. All authors contributed to the article and approved the submitted version.
